# Tumor Treating Fields and the Glioblastoma Microenvironment: Mechanistic Convergences with Radiotherapy

**DOI:** 10.3390/cancers18132069

**Published:** 2026-06-25

**Authors:** Flavio Donnini, Giuseppe Battaglia, Salvatore Chibbaro, Francesco Marampon, Giuseppe Minniti, Paolo Tini

**Affiliations:** 1 Unit of Radiation Oncology, Department of Medicine, Surgery and Neurosciences, University of Siena, 53100 Siena, Italy; 2 Unit of Neurosurgery, Department of Medicine, Surgery and Neurosciences, University of Siena, 53100 Siena, Italy; 3 Radiation Oncology, Policlinico Umberto I, Department of Radiological, Oncological and Pathological Sciences, ‘Sapienza’ University of Rome, 00185 Rome, Italy; 4 IRCCS Neuromed, 86077 Pozzilli, Italy

**Keywords:** glioblastoma, tumor treating fields, tumor microenvironment, radiotherapy, cGAS-STING pathway, immunogenic cell death, glioma stem cells, blood–brain barrier

## Abstract

Glioblastoma (GBM) remains the most lethal primary brain tumor in adults, with a median overall survival of approximately 15–20 months despite multimodal treatment including surgery, chemoradiation, and Tumor Treating Fields (TTFields). While the survival benefit of TTFields was established by the EF-14 phase III trial, their biological effects extend well beyond the canonical anti-mitotic mechanism and encompass extensive interactions with the GBM tumor microenvironment (TME). This review provides an integrated mechanistic analysis of TTFields–TME interactions in GBM, with a distinctive focus on their convergence with radiotherapy. We examine how TTFields activate innate immune sensing through cGAS/STING and AIM2 inflammasome pathways, drive immunogenic cell death, reprogram tumor-associated macrophages, and prime adaptive T cell responses. We further address TTFields effects on glioma stem cells, blood–brain barrier permeability, and intracellular signaling governing invasion, angiogenesis, and autophagy. Critically, we develop the mechanistic and clinical case for TTFields-radiotherapy combinations, highlighting convergent mechanisms of DNA repair impairment, mitotic catastrophe, and innate immune activation. Practical considerations for concurrent clinical implementation are discussed alongside a research agenda centered on optimal timing, hypofractionation, and predictive biomarkers. Available evidence—largely preclinical—suggests that TTFields may act as a TME-remodeling platform whose potential is most likely to be realized through mechanistically informed combinations.

## Simple Summary

Glioblastoma is the most aggressive primary brain tumor in adults and remains largely incurable despite surgery, radiotherapy, and chemotherapy. Tumor Treating Fields (TTFields) are alternating electric fields delivered through scalp electrodes that improve survival in selected patients, but their biological effects extend beyond disruption of tumor cell division. This review explains how TTFields may interact with the glioblastoma tumor microenvironment by activating immune-sensing pathways, affecting glioma stem cells, increasing blood–brain barrier permeability, and modulating signaling pathways involved in invasion, angiogenesis, and survival. Particular emphasis is placed on the convergence between TTFields and radiotherapy, since both treatments may impair DNA damage repair and activate overlapping immune mechanisms. Understanding these interactions may support rational combinations of TTFields with radiotherapy, immunotherapy, and targeted agents, and may guide future biomarker-driven clinical trials.

## 1. Introduction

Glioblastoma (GBM) remains the most lethal primary brain tumor in adults, with a median overall survival of approximately 15–20 months despite maximal therapeutic efforts. The current standard of care, established by Stupp et al. in 2005, consists of maximal safe surgical resection followed by concurrent radiotherapy and temozolomide (TMZ), with subsequent adjuvant TMZ chemotherapy [1]. Despite decades of clinical investigation, this paradigm has yielded only marginal improvements in survival, and virtually all patients experience tumor recurrence within months of completing initial treatment [1,2].

Tumor Treating Fields (TTFields) represent a locoregional, non-invasive treatment modality that delivers low-intensity (1–3 V/cm), intermediate-frequency (200 kHz for GBM) alternating electric fields to the tumor region via transducer arrays placed on the scalp. The pivotal EF-14 phase III randomized controlled trial demonstrated that adding TTFields to maintenance TMZ significantly improved both progression-free survival (6.7 vs. 4.0 months) and overall survival (20.9 vs. 16.0 months) compared to TMZ alone, leading to regulatory approval of the device as part of the standard-of-care regimen for newly diagnosed GBM [2]. Despite this established clinical benefit, the biological mechanisms underlying the efficacy of TTFields extend considerably beyond the classically described anti-mitotic effects on tubulin polymerization and septin localization, and are increasingly recognized as multidimensional and microenvironment-dependent [3,4].

The tumor microenvironment (TME) of GBM is a profoundly immunosuppressive and biologically complex ecosystem, characterized by extensive infiltration of tumor-associated macrophages and microglia (TAMs), myeloid-derived suppressor cells (MDSCs), regulatory T cells (Tregs), a population of therapy-resistant glioma stem cells (GSCs), and a dysfunctional vasculature constrained by the blood–brain barrier (BBB) [5]. This immunosuppressive milieu, compounded by the unique immune privilege of the central nervous system, renders GBM a prototypical “cold tumor” refractory to immune checkpoint inhibition and largely inaccessible to systemic drug delivery [5,6]. Any treatment strategy capable of meaningfully reshaping the GBM TME, therefore, holds exceptional translational relevance, both as a standalone approach and as a platform for rational therapeutic combinations.

Accumulating preclinical evidence now indicates that TTFields exert a broad spectrum of effects on the GBM TME, operating through mechanisms that are distinct from, yet synergistic with, those of standard chemoradiation. These include activation of innate immune sensing pathways via cytosolic DNA accumulation and cGAS/STING signaling, induction of immunogenic cell death through inflammasome activation, modulation of adaptive immune responses, direct targeting of GSC self-renewal, reversible disruption of BBB integrity, and perturbation of intracellular signaling cascades governing tumor cell motility and survival [3,4,7,8]. Critically, these TME-modulatory effects position TTFields as a potential immunological sensitizer—and, from a radiation oncology perspective, as a mechanistic convergent partner for radiotherapy.

Several reviews have addressed aspects of TTFields biology in GBM. Guo et al. provided a comprehensive overview of TTFields mechanisms and clinical data, including initial observations on immune modulation [3]. Liu et al. examined TTFields in the context of combination therapeutic strategies [4]. Fuso Nerini et al. offered a pan-tumor perspective on TTFields interactions with the TME across solid tumor histologies [8]. None of these, however, has examined the TTFields–TME interaction in GBM through an integrated, radiation oncology-centric lens. Such a perspective would systematically incorporate the most recent immunological, translational, and clinical data—cGAS/STING activation, GSC-specific DDR vulnerability, BBB modulation, and the emerging trial landscape—within a single mechanistic framework bearing directly on concurrent clinical implementation and trial design.

The present narrative review addresses this gap. Specifically, it differs from prior publications in three respects. First, unlike reviews focused on TTFields mechanisms [3] or combination strategies [4], it examines TTFields-TME interactions through an integrated radiation oncology-centric framework, treating the TME not as a background context but as the primary biological substrate through which TTFields and radiotherapy interact. Second, it incorporates the most recent translational and clinical evidence—including cGAS/STING pathway activation data from the 2-THE-TOP trial [9], GSC-specific DDR vulnerability [10], reversible BBB modulation in human models [11], and the TRIDENT phase 3 trial [12]—into a unified mechanistic narrative that was not available to earlier reviewers. Third, it proposes a structured translational research agenda with specific testable hypotheses and candidate biomarkers, rather than limiting open questions to a descriptive enumeration. We provide an integrated analysis of the mechanisms by which TTFields reshape the GBM TME, with particular focus on innate and adaptive immune modulation, GSC biology, BBB permeability, and intracellular signaling. We then examine in depth the mechanistic and clinical convergences between TTFields and radiotherapy, discussing radiobiological synergies, immunological co-activation, and practical considerations for concurrent delivery. Finally, we outline open questions and propose a framework for the rational design of TTFields-based combination strategies in GBM.

## 2. TTFields: Physical Principles and Established Mechanisms in GBM

TTFields are alternating electric fields delivered at low intensity (1–3 V/cm) and intermediate frequency, with 200 kHz identified as the optimal frequency for GBM cells based on their size and dielectric properties [13]. The electric fields are generated by a portable medical device and transmitted to the tumor region via transducer arrays—adhesive patches containing ceramic disk electrodes—applied directly to the shaved scalp and worn continuously for a minimum of 18 h per day [2,13]. Two pairs of arrays are positioned perpendicularly to one another to deliver fields in alternating directions, ensuring that dividing cells oriented in any plane are exposed to therapeutic field intensities throughout the treatment period [13,14].

The anti-mitotic mechanism of TTFields was first characterized by Kirson et al., who demonstrated that alternating electric fields at specific frequencies preferentially disrupt dividing cells through two sequential processes [13]. During metaphase, TTFields interact with the dipole moment of α/β-tubulin heterodimers, impairing microtubule polymerization and disrupting mitotic spindle assembly. During late anaphase and telophase, the geometrically non-uniform electric field generated at the narrowing cleavage furrow drives polar intracellular constituents—including septin proteins critical for cytokinesis—toward the furrow via dielectrophoresis, leading to aberrant mitotic exit, formation of aneuploid daughter cells, and ultimately cell death [13,14,15].

Beyond their canonical anti-mitotic activity, TTFields exert a broader spectrum of biological effects that have progressively been characterized in preclinical GBM models. These include: disruption of primary cilia, which are aberrantly retained in GSCs and function as oncogenic signaling hubs for pathways including Sonic Hedgehog; induction of nuclear envelope disruption leading to cytoplasmic extrusion of chromatin fragments and formation of micronuclei; impairment of DNA double-strand break repair, particularly through downregulation of homologous recombination mediators; perturbation of autophagy flux; and inhibition of cell migration and invasion through reorganization of the actin cytoskeleton and focal adhesion complexes [14,15,16]. Several of these mechanisms directly interface with the TME and with innate immune sensing pathways, forming the mechanistic substrate for the immunomodulatory effects of TTFields that are examined in detail in subsequent sections of this review.

The clinical efficacy of TTFields is compliance-dependent: in a subgroup analysis of the EF-14 trial, patients achieving device compliance exceeding 90% demonstrated a median overall survival of 24.9 months and a five-year survival rate of 29.3% [17]. This dose–response relationship between field exposure duration and clinical outcome is conceptually analogous to the fractionation-response relationship in radiotherapy and carries direct implications for the scheduling of TTFields relative to radiation treatment, although this compliance-stratified association derives from a post hoc, non-randomized subgroup analysis and is subject to the methodological caveats discussed in Section 11.

The present review does not aim to provide an exhaustive account of TTFields mechanisms and clinical efficacy in GBM, topics that have been comprehensively addressed in dedicated publications [3,4,16]. Rather, the following sections focus specifically on those mechanisms operating at the interface between TTFields and the TME—particularly those with relevance to innate and adaptive immune activation, GSC biology, BBB permeability, and intracellular signaling—and on their translational implications for radiation oncology.

## 3. The GBM Tumor Microenvironment: A Brief Conceptual Framework

Before examining how TTFields interact with each component of this ecosystem, a brief operational definition of the GBM TME is warranted. GBM is characterized by one of the most immunosuppressive and therapeutically recalcitrant tumor microenvironments described across human malignancies [5,6]. Understanding the architecture of the GBM TME is essential to appreciate both the mechanisms by which TTFields interact with it and the translational implications of those interactions for combination treatment strategies.

At the cellular level, the GBM TME is dominated by non-neoplastic components that collectively enforce immunosuppression and sustain tumor growth. Tumor-associated macrophages and microglia (TAMs) constitute the most abundant immune cell population within GBM, comprising up to 30–50% of the total tumor mass [5]. Derived from both resident microglia and peripheral bone marrow-derived monocytes recruited across a disrupted BBB, TAMs in GBM predominantly adopt an alternatively activated (M2-like) phenotype characterized by secretion of immunosuppressive mediators including interleukin (IL)-10, transforming growth factor-β (TGF-β), and vascular endothelial growth factor (VEGF), thereby promoting tumor angiogenesis, immune evasion, and therapeutic resistance [5,18]. Alongside TAMs, MDSCs and Tregs further suppress effector T cell function through multiple non-redundant mechanisms, including upregulation of immune checkpoint ligands such as programmed death-ligand 1 (PD-L1) and cytotoxic T-lymphocyte-associated protein 4 (CTLA-4) co-stimulatory pathway interference [6]. Natural killer (NK) cell infiltration is sparse and functionally impaired. The net result is a profoundly immunologically “cold” tumor that has proven largely refractory to immune checkpoint inhibitor monotherapy in clinical trials [6].

A second defining feature of the GBM TME is the presence of a GSC population, organized within specialized niches—principally the perivascular niche, the hypoxic/necrotic niche, and the invasive margin—that sustain self-renewal, drive tumor repopulation following therapy, and actively remodel the surrounding immune microenvironment [18]. GSCs secrete immunosuppressive factors, recruit pro-tumoral TAMs, contribute to vascular pericyte populations that reinforce the blood-tumor barrier, and exhibit intrinsic resistance to both radiotherapy and chemotherapy through upregulation of DNA damage response pathways and anti-apoptotic mechanisms [18].

The BBB and its pathologically altered counterpart in GBM, the blood-tumor barrier (BTB), represent an additional layer of complexity. While partially disrupted in regions of contrast enhancement visible on MRI, the BBB remains largely intact in the infiltrative, non-enhancing tumor margin—the zone from which recurrence most commonly originates—limiting the penetration of systemic therapeutic agents and restricting immune cell trafficking [5,6].

Finally, the GBM TME is profoundly shaped by metabolic and physicochemical factors, including intratumoral hypoxia driven by aberrant vasculature, elevated interstitial fluid pressure, and acidosis, all of which further polarize TAMs toward immunosuppressive phenotypes, promote GSC maintenance, and confer radioresistance [5]. This multi-layered immunosuppressive ecosystem defines the context in which TTFields operate.

## 4. TTFields and Innate Immunity: cGAS/STING and Inflammasome Activation

The recognition of cytosolic DNA as a danger signal is a fundamental mechanism of innate immune surveillance that has emerged in the last decade as a central determinant of antitumor immunity. Two non-redundant cytosolic DNA sensors, the cyclic GMP-AMP synthase (cGAS)–stimulator of interferon genes (STING) axis and the absent in melanoma 2 (AIM2) inflammasome, jointly translate the presence of mislocalized nuclear or mitochondrial DNA into proinflammatory and immunogenic responses [7]. Engagement of cGAS by cytosolic double-stranded DNA catalyzes the synthesis of 2′3′-cyclic GMP-AMP (cGAMP), which binds and activates STING at the endoplasmic reticulum membrane. The downstream cascade—through TANK-binding kinase 1 (TBK1) and interferon regulatory factor 3 (IRF3)—drives transcription of type I interferons (IFN-α/β) and interferon-stimulated genes that promote dendritic cell (DC) maturation, antigen cross-presentation, and CD8^+^ T cell priming [7]. In parallel, AIM2 oligomerizes upon binding cytosolic dsDNA and recruits ASC and pro-caspase-1 to assemble an inflammasome. This complex matures pro-interleukin (IL)-1β and pro-IL-18 and cleaves gasdermin D, the executioner of pyroptosis—a lytic, immunogenic mode of cell death characterized by plasma membrane permeabilization and release of damage-associated molecular patterns (DAMPs) [7]. In the GBM TME, where both intrinsic immunogenicity and antigen presentation are profoundly suppressed, pharmacological or physical strategies capable of activating these sensors represent a particularly attractive route toward immunological reprogramming.

A key study by Chen et al. provided the first integrated mechanistic demonstration that TTFields engage both cGAS/STING and AIM2 inflammasome signaling in GBM [7]. Using a combination of patient-derived GBM cell lines and a syngeneic orthotopic murine model in immunocompetent C57BL/6 hosts, the authors showed that TTFields application at 200 kHz induces extensive nuclear envelope rupture, with the consequent formation of cytosolic micronuclei containing genomic dsDNA. These mislocalized DNA species function as the proximal trigger of dual innate immune sensing: cGAS binds cytosolic dsDNA and activates STING-dependent type I IFN production, while AIM2 oligomerizes on the same substrate to drive caspase-1 activation, IL-1β/IL-18 secretion, and gasdermin D-mediated pyroptotic cell death [7]. Genetic ablation or pharmacological inhibition of cGAS, STING, or AIM2 in tumor cells abrogated the downstream cytokine response and, critically, the antitumor immunity observed in vivo, demonstrating that the immune-modulating activity of TTFields is dependent on these innate sensors [7]. In treated animals, intratumoral TTFields delivery promoted DC maturation, increased infiltration of CD4^+^ and CD8^+^ T cells into the brain tumor, and prolonged survival in an immune-dependent manner—effects that were lost in immunodeficient hosts [7]. In this model, these findings position TTFields not merely as an anti-mitotic modality but as a candidate inducer of innate immune activation within the brain TME, although this demonstration rests on a single integrated preclinical study and awaits independent confirmation. Importantly, the dual cGAS/STING and AIM2 mechanism reported by Chen et al. [7] has not, to our knowledge, been independently replicated in other GBM models or laboratories, nor has a contradictory dataset yet been published. Several of the principal downstream conclusions drawn in this review—tumor-cell-intrinsic dual inflammasome activation, the immune-dependent survival benefit, and clonotypic T cell expansion—derive substantially from this single source and should therefore be regarded as provisional pending independent confirmation.

**Figure 1:**
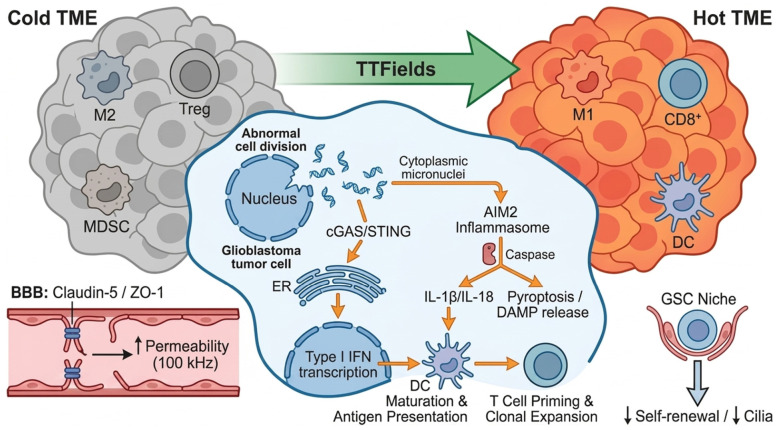
TTFields-induced remodeling of the glioblastoma tumor microenvironment. TTFields disrupt the nuclear envelope of dividing glioblastoma cells, generating cytoplasmic micronuclei that activate cGAS/STING signaling (→ type I IFN transcription) and the AIM2 inflammasome (→ IL-1β/IL-18 release and pyroptosis). Downstream DC maturation and CD8^+^ T cell priming promote features associated with a shift from an immunosuppressive “cold” state (M2 macrophages, Tregs, MDSCs) toward an immunologically active “hot” state. TTFields additionally increase BBB permeability via claudin-5/ZO-1 disruption (100 kHz) and reduce GSC self-renewal and primary cilia expression. Abbreviations: AIM2, absent in melanoma 2; BBB, blood–brain barrier; cGAS, cyclic GMP-AMP synthase; DC, dendritic cell; GSC, glioma stem cell; IFN, interferon; MDSC, myeloid-derived suppressor cell; STING, stimulator of interferon genes; TME, tumor microenvironment; Treg, regulatory T cell; TTFields, Tumor Treating Fields; ZO-1, zonula occludens-1.

Complementary evidence from non-GBM tumor models has reinforced the concept that TTFields elicit immunogenic cell death (ICD). Voloshin and colleagues demonstrated, across multiple cancer cell line systems, that TTFields treatment induces the release of HMGB1 and ATP and the cell-surface exposure of calreticulin—the canonical molecular hallmarks of ICD—and promotes DC engulfment of treated cancer cells with subsequent DC maturation in vitro [19]. In syngeneic in vivo models, combining TTFields with anti-PD-1 therapy resulted in increased intratumoral leukocyte infiltration and superior tumor control compared to either modality alone [19]. While these data were generated in lung and ovarian cancer models rather than GBM, they are consistent with the GBM-specific observations of Chen et al. [7] and suggest a mechanism whereby TTFields promote features of immunogenic cell death; whether this amounts to functional in situ vaccination in GBM remains to be established. This distinction is not merely semantic: functional immunogenic cell death, and, in particular, a productive in situ vaccination effect, has not been directly demonstrated in GBM models, and two GBM-intrinsic features may attenuate it relative to the lung and ovarian systems in which the ICD hallmarks were originally characterized. First, GBM exhibits low baseline immunogenicity, with a comparatively modest tumor mutational and neoantigen burden. Second, the GBM TME is densely infiltrated by regulatory T cells and other immunosuppressive populations. Either feature could blunt the conversion of DAMP release into effective antigen cross-presentation and durable T cell priming, so that the immunogenic potential inferred from non-GBM models should not be assumed to transfer quantitatively to glioblastoma. The integrated mechanistic cascade by which TTFields remodel the GBM TME, encompassing innate immune sensing, adaptive T cell priming, BBB modulation, and GSC targeting, is summarized in Figure 1.

Beyond the direct effects on tumor cells, TTFields exposure has been shown to reprogram the phenotype of TAMs themselves. In a recent study by Kan and colleagues, TTFields application in tumor cell–macrophage co-culture systems shifted macrophages away from an M2-like, immunosuppressive phenotype toward an M1-like, proinflammatory state, with concurrent activation of GEF-H1/NF-κB/MyD88 signaling and upregulation of nitric oxide synthase, TNF-α, and IL-12 [20]. Although this work was performed in non-small cell lung cancer models using peripheral, monocyte-derived macrophages—whose developmental origin and activation biology differ substantially from those of brain-resident microglia, which constitute a major component of the GBM myeloid compartment—and direct evidence in GBM-associated microglia and macrophages remains limited, the implication for the GBM TME, dominated as it is by M2-like TAMs, is biologically significant and warrants dedicated investigation in glioma-specific models. Whether TTFields can repolarize ontogenetically distinct, brain-resident microglia—as opposed to recruited macrophages—is an explicit and currently unresolved question.

The convergent picture that emerges is one of a multilayered innate immune activation: TTFields disrupt the nuclear envelope of dividing GBM cells, expose genomic DNA to the cytosol, simultaneously engage cGAS/STING and AIM2 inflammasome signaling, drive pyroptotic and immunogenic cell death with DAMP release, mature local DCs, and may additionally repolarize the TAM compartment toward proinflammatory phenotypes. Each of these effects directly counters core features of the GBM immunosuppressive landscape outlined in the preceding section. Critically, several of these mechanisms—cytosolic DNA accumulation, micronucleus formation, cGAS/STING engagement, and ICD—are also induced, through partly overlapping and partly distinct routes, by ionizing radiation.

Important caveats remain. Most preclinical data on TTFields-induced innate immune activation derive from cell line and syngeneic mouse models, which only partially recapitulate the cellular complexity and immune privilege of the human GBM TME. The kinetics of cGAS/STING activation in patient tumors, the relative contributions of tumor-intrinsic versus stromal STING signaling, and the potential for chronic STING engagement to induce immune exhaustion or paradoxical tolerogenic effects are all open questions. This caveat is central because it reflects a fundamental duality of the pathway: the same cGAS/STING axis that drives type I interferon production and T cell priming can, under sustained or chronic engagement, instead favor tolerogenic and T-cell-exhaustion programs, so that the net immunological direction of TTFields-induced STING signaling is not predetermined. This tension is developed as a controversy in Section 12.1. Distinguishing productive from pathological STING output in future studies will likely require temporal readouts—acute, self-limited type I IFN induction versus chronic, NF-κB-biased signaling—rather than single-timepoint measurements. Furthermore, the efficacy of TTFields-driven immune activation in patients with pre-existing lymphopenia—a frequent consequence of corticosteroid use and prior chemoradiotherapy—has not been systematically characterized. These questions define a priority research agenda discussed in Section 11 and Section 12.

## 5. TTFields and Adaptive Immunity: T Cell Priming, Clonal Expansion, and Clinical Translation

The innate immune activation triggered by TTFields, described in the preceding section, does not operate in isolation but serves as the proximal driver of downstream adaptive immune responses. The mechanistic sequence—nuclear envelope rupture, cytosolic DNA sensing, type I interferon production, and DC maturation—constitutes a classical pathway for antigen cross-presentation and CD8^+^ T cell priming, with directly measurable consequences for the adaptive immune landscape of GBM.

In the syngeneic orthotopic GBM model employed by Chen et al., TTFields treatment resulted in significantly increased infiltration of both CD4^+^ and CD8^+^ T cells into the tumor parenchyma, a finding that was dependent on intact cGAS/STING and AIM2 signaling in tumor cells [7]. Single-cell RNA sequencing of tumor-infiltrating lymphocytes from treated animals revealed evidence of clonotypic T cell expansion, consistent with antigen-driven selection rather than non-specific bystander activation [7]. Importantly, tumor rechallenge experiments in animals that had achieved long-term survival following TTFields treatment demonstrated durable immunological memory, with complete rejection of rechallenge in many animals—a result that would not be expected from a treatment acting exclusively through direct tumor cell killing [7]. Collectively, these preclinical data are consistent with the hypothesis that TTFields-treated GBM cells may act as an in situ vaccination stimulus: a source of tumor-associated antigens released in an immunostimulatory, DAMP-rich, type I IFN-polarized context that fulfills several of the classical requirements for productive T cell priming. As this interpretation derives largely from a single syngeneic model, it should be regarded as hypothesis-generating.

The type I interferon response induced by cGAS/STING activation also has secondary consequences for the adaptive immune landscape that create both opportunity and potential resistance. On one hand, IFN-α/β signaling promotes DC cross-presentation efficiency, NK cell activation, and the upregulation of MHC-I on tumor cells, collectively enhancing the visibility of GBM cells to the adaptive immune system [7,19]. On the other hand, sustained type I IFN signaling and the inflammatory remodeling of the TME are well-established inducers of adaptive immune resistance, including transcriptional upregulation of PD-L1 on both tumor cells and infiltrating myeloid populations through IFN-γ-driven JAK/STAT signaling [6]. This mechanistic duality—whereby TTFields simultaneously activate T cell priming and induce immune checkpoint upregulation—constitutes the core immunological rationale for combining TTFields with programmed death-1 (PD-1) or PD-L1 blockade.

The translational validation of this rationale is provided by the 2-THE-TOP phase 2 trial (NCT03405792), which evaluated the combination of TTFields, adjuvant temozolomide, and pembrolizumab in 31 patients with newly diagnosed GBM [9]. Peripheral blood mononuclear cell sequencing performed before pembrolizumab initiation—allowing attribution of immune effects to TTFields alone—revealed robust T cell activation via the type I interferon trajectory in 11 of 12 evaluable patients, with a strong correlation between the magnitude of cGAS/STING-dependent IFN signaling and T cell receptor αβ clonal expansion (Spearman coefficient r = −0.80, *p* = 0.014) [9]. Clinically, patients with larger residual tumor burden at the time of TTFields initiation—hypothesized to provide a more abundant antigen source for in situ vaccination—showed disproportionately greater improvements in progression-free and overall survival relative to matched historical controls compared to patients who had undergone maximal resection [9]. These findings provide among the first clinical, correlative signals consistent with an in situ immunizing effect of TTFields in GBM patients. They should, however, be interpreted cautiously: the immune correlates derive from a single-arm phase 2 study with only 12 evaluable patients for the pre-pembrolizumab analysis, and the survival comparison by residual tumor burden relied on matched historical controls rather than a randomized design. With these caveats, the data provided the mechanistic rationale for the registrational-intent EF-41/KEYNOTE-D58 phase 3 trial [21]. Notably, these immune correlates were not stratified by concurrent corticosteroid exposure, a recognized confounder of TTFields-driven immune activation that we revisit in Section 11.

Despite this encouraging translational trajectory, several limitations specific to the GBM immune contexture constrain the magnitude of adaptive immune responses achievable with TTFields, even in combination with immune checkpoint inhibitors (ICI). First, GBM patients typically present with profound systemic lymphopenia, compounded by corticosteroid-dependent immunosuppression during and after chemoradiotherapy, which substantially reduces the peripheral T cell pool available for tumor-antigen-driven expansion [6]. Second, the BBB and blood-tumor barrier restrict the trafficking of activated effector T cells into the tumor parenchyma, even when T cell priming in the periphery is successful [5,6]. Third, within the GBM TME, MDSCs and Tregs maintain potent suppression of effector T cell function through multiple non-redundant mechanisms, including arginase-1-mediated arginine depletion, TGF-β secretion, and IL-10-mediated inhibition—mechanisms not directly targeted by PD-1 blockade or by TTFields as currently understood [5,6]. Fourth, the T cell exhaustion phenotype observed in glioblastoma-infiltrating lymphocytes, characterized by co-expression of multiple inhibitory receptors including PD-1, TIM-3, and LAG-3, may limit the durability of responses achieved through PD-1 blockade alone [6]. These considerations suggest that the full exploitation of TTFields-induced adaptive immunity in GBM will require combination strategies that address each of these resistance layers simultaneously.

## 6. TTFields and Glioma Stem Cells

Glioma stem cells occupy a unique position in the biology of GBM that makes them simultaneously a principal driver of therapeutic failure and a compelling target for biologically informed combination strategies. Defined operationally by their capacity for self-renewal, multi-lineage differentiation, and tumor propagation upon serial transplantation, GSCs constitute a minor but functionally dominant cellular fraction within the tumor that is responsible for initiating tumor growth, sustaining clonal evolution, and repopulating the tumor parenchyma following cytotoxic treatment [18]. GSCs are organized within specialized niches that render them particularly difficult to eradicate with locoregional therapies delivering inhomogeneous field or dose distributions [5,18]. In the perivascular niche, GSCs remain close to endothelial cells and pericytes to sustain self-renewal; in the hypoxic/necrotic niche, low oxygen tension maintains stemness through HIF-1α-dependent transcription; and the invasive margin is the principal source of post-treatment repopulation. Critically, GSCs are not passive bystanders within the TME but active remodelers of it: they recruit and polarize TAMs through secretion of periostin and WNT family member 1-inducible signaling protein 1, directly suppressing antitumor immunity and reinforcing the immunosuppressive ecosystem described in the preceding sections [5].

Preclinical evidence demonstrates that TTFields exert direct anti-proliferative and cytotoxic effects on GSC populations. In a foundational study by Silginer and colleagues, TTFields application at 200 kHz induced cell death, aberrant mitotic features, and impaired migration not only in established GBM cell lines but also in patient-derived glioma-initiating cells maintained under stem-cell conditions [22]. Cell death in this model was notably independent of classical caspase-mediated apoptosis and instead involved autophagy and necroptosis—death modalities with distinct immunological consequences relevant to the broader TME-modulatory framework of this review [22]. These findings suggest that the direct anti-tumor activity of TTFields extends beyond the rapidly cycling, differentiated bulk tumor cell population to encompass the slower-cycling, therapy-resistant GSC compartment, although the degree of this effect is likely influenced by the inherently lower proliferative rate of quiescent GSCs in vivo.

A distinctive interaction between TTFields and GSC biology involves the primary cilium, a microtubule-based sensory organelle that is aberrantly retained and overexpressed in GBM cells relative to normal brain parenchyma. Primary cilia in GBM function as oncogenic signaling platforms, transducing pro-growth stimuli through the Sonic Hedgehog (SHH)/GLI axis, receptor tyrosine kinase pathways, and G-protein-coupled receptors, all of which contribute to GSC self-renewal and chemoresistance [10,23]. TTFields disrupt primary cilia integrity in GBM cells in vitro and ex vivo, an effect that converges with the anti-mitotic mechanism of microtubule destabilization and appears to sensitize GSCs to both TMZ and TTFields-mediated killing [23]. Importantly, TMZ paradoxically promotes ciliogenesis in GBM cells as a resistance adaptation—a mechanism that TTFields may counteract by directly ablating newly formed cilia, providing one molecular explanation for the cooperative interaction between the two modalities that extends beyond simple additive cytotoxicity [23].

The interaction between TTFields and the DNA damage response (DDR) in GSCs has been systematically characterized by Vanderlinden and colleagues using a patient-derived living biobank of more than 110 GSC models incorporating multi-region sampling to capture intratumoral heterogeneity [10]. These models, representing the therapy-resistant, infiltrative margin of surgically resected GBMs, demonstrated significant synergistic growth inhibition when TTFields were combined with PARP inhibitors (olaparib, niraparib) or ATR inhibitors—agents targeting the DDR pathways that GSCs rely upon to survive replication stress and radiation-induced DNA damage [10]. The mechanistic basis for this synergy resides in the observation that TTFields impair homologous recombination-mediated DNA repair, creating a conditional DDR vulnerability that is selectively exploited by DDR inhibitors and that recapitulates, in the GSC compartment, the broader TTFields-induced suppression of the FA-BRCA-RAD51 network demonstrated in non-small cell lung cancer and other tumor models [24,25]. This TTFields-induced HR deficiency in the GSC compartment is continuous with the radiosensitization effects described in Section 9.1, suggesting that the DDR vulnerability created by TTFields operates across both the differentiated tumor bulk and the therapy-resistant stem cell fraction.

From the perspective of TME remodeling, the capacity of TTFields to directly target GSCs adds a further dimension to its immunological effects. GSCs are a primary source of immunosuppressive signals within the GBM TME, including VEGF-driven angiogenesis that maintains vascular barrier function, TGF-β secretion that polarizes TAMs toward M2 phenotypes, and PD-L1 surface expression that directly suppresses T cell activity [5,18]. Therapeutic strategies that reduce GSC abundance or impair GSC immune-modulatory functions therefore have the potential to reinforce the innate and adaptive immune activation described in Section 4 and Section 5, creating a mechanistic positive feedback loop: TTFields kill GSCs and release tumor antigens under immunostimulatory conditions, while simultaneously reducing the GSC-derived immunosuppressive pressure that would otherwise blunt the resulting T cell response. Whether this synergism is quantitatively meaningful in the human GBM TME, given the complexity and redundancy of immunosuppressive mechanisms, remains an open empirical question and a priority for future investigation.

## 7. TTFields and the Blood–Brain Barrier

The blood–brain barrier constitutes one of the most formidable obstacles to effective pharmacological treatment of GBM. Maintained by the tight junction complexes of specialized brain capillary endothelial cells—principally claudin-5, occludin, and the cytoplasmic scaffolding protein zonula occludens-1 (ZO-1)—in concert with astrocyte end-feet, pericytes, and a non-cellular basement membrane, the BBB restricts paracellular and transcellular transport of the majority of systemically administered therapeutic agents [5]. While the contrast-enhancing core of GBM exhibits partial barrier disruption visible on MRI, the infiltrative non-enhancing margin—the compartment most relevant to recurrence—retains substantial barrier integrity, limiting the delivery of both cytotoxic and targeted agents to the tumor cells most likely to escape local therapy [5,6]. The capacity of TTFields to modulate BBB permeability, therefore, represents a potentially high-impact TME interaction with direct drug delivery implications.

The first systematic characterization of TTFields effects on the BBB was provided by Salvador and colleagues, who demonstrated that TTFields application at 100 kHz induces reversible, frequency-dependent disruption of BBB integrity in murine microvascular cerebellar endothelial cells (cerebEND) in vitro and in healthy rat brain in vivo [26]. The molecular mechanism involves Rho kinase (ROCK)-mediated phosphorylation of claudin-5, leading to delocalization of this critical tight junction protein from the cell membrane to the cytoplasm, with concurrent displacement of ZO-1 and occludin [26]. Quantitatively, TTFields treatment resulted in a 65% reduction in transendothelial electrical resistance—the gold-standard in vitro measure of barrier integrity—and a significant increase in paracellular permeability to 4 kDa molecules [26]. In tumor-bearing rats, a combination of TTFields with paclitaxel, a chemotherapeutic agent that is normally excluded by the intact BBB, produced superior tumor volume reduction compared to either treatment alone, providing proof-of-concept for TTFields as a non-invasive CNS drug delivery enhancer [26].

The reversibility of this BBB effect is mechanistically important from both a therapeutic and safety perspective. Cell morphology and tight junction protein localization begin to recover within 48 h of TTFields cessation, with complete functional recovery of barrier integrity within 96 h in both in vitro and in vivo models [26]. Serial dynamic contrast-enhanced MRI (DCE-MRI) in treated rats corroborated these kinetics, showing gadolinium accumulation in brain parenchyma during active TTFields delivery that resolved to baseline after treatment discontinuation [26]. This reversibility distinguishes TTFields-induced BBB modulation from pathological barrier breakdown, suggesting a therapeutically exploitable, temporally controlled permeability window rather than a sustained compromise of neuroprotective function.

Translation of these findings to human-relevant models was subsequently validated by Salvador and colleagues using a three-dimensional co-culture BBB model composed of primary human brain microvascular endothelial cells and immortalized human pericytes [11]. TTFields at 100 kHz for 72 h increased permeability in this human-derived system, with barrier recovery occurring at 48 h post-treatment—earlier than in the murine cerebEND model, suggesting potential species-specific differences in tight junction regulation that merit consideration in clinical translation [11]. Clinical in-human evidence was provided by Iv and colleagues, who employed perfusion MRI to demonstrate increased relative cerebral blood volume and BBB permeability in GBM patients receiving TTFields at the clinically approved frequency of 200 kHz, establishing that the BBB-modulatory effect is observable even at frequencies optimized for anti-mitotic rather than BBB-disrupting activity [27].

Two considerations temper the translational enthusiasm surrounding TTFields-induced BBB modulation. First, the frequency optimum for BBB disruption (100 kHz) differs from that for anti-tumor efficacy in GBM (200 kHz), creating an inherent tension between maximizing BBB permeability and maintaining anti-mitotic activity at currently approved device settings [26,27]. Second, the clinical significance of the observed BBB permeability changes—whether sufficient to meaningfully increase parenchymal concentrations of co-administered therapeutic agents—has not yet been demonstrated in prospective pharmacokinetic studies in GBM patients. These open questions define a priority area for translational investigation, particularly in the context of combining TTFields with agents that are otherwise BBB-restricted, including targeted therapies, antibody-drug conjugates, and immunological agents.

## 8. Intracellular Signaling Crossroads: Integrins, Mitosis, and Autophagy

Beyond the immunological and stem cell-related effects described in the preceding sections, TTFields interact with a network of intracellular signaling pathways governing GBM cell motility, invasion, angiogenesis, and stress responses. These interactions extend the mechanistic reach of TTFields into the cellular infrastructure that sustains tumor maintenance and therapeutic resistance and carry TME-level consequences that are directly relevant to the integrated framework presented in this review.

**Integrin and focal adhesion signaling.** Cell migration and invasion in GBM are orchestrated by the coordinated activity of the actin cytoskeleton, microtubule dynamics, and the focal adhesion complexes that mechanically link the intracellular cytoskeleton to the extracellular matrix via integrin receptors. Voloshin and colleagues demonstrated that TTFields exposure disrupts GBM cell motility through a pathway involving GEF-H1, a microtubule-associated Rho guanine nucleotide exchange factor that normally releases RhoA upon microtubule depolymerization [28]. TTFields-induced microtubule destabilization activates GEF-H1, which in turn engages the RhoA/ROCK/myosin light chain kinase axis, leading to aberrant cytoskeletal contractility, disrupted focal adhesion turnover, and impaired lamellipodia formation [28]. The net result is a significant, concentration-dependent reduction in directional cell migration and invasion, quantified by wound-healing and transwell assays across multiple GBM cell lines. This anti-invasive effect operates independently of the anti-mitotic mechanism, suggesting that TTFields simultaneously impair two cardinal determinants of GBM lethality—uncontrolled proliferation and diffuse infiltration—through distinct but mechanistically linked cytoskeletal targets.

Complementary data from Kim and colleagues extended this picture to encompass the transcriptional regulation of invasion and angiogenesis [29]. TTFields suppressed GBM cell migration and invasion in association with dysregulation of epithelial-to-mesenchymal transition (EMT)-related genes, including downregulation of vimentin and upregulation of E-cadherin, effects consistent with a partial mesenchymal-to-epithelial phenotypic shift [29]. At the signaling level, TTFields inhibited NF-κB transcriptional activity—a master regulator of inflammatory gene expression, invasion, and resistance to apoptosis—and reduced phosphorylation of MAPK and PI3K/AKT pathway intermediates [29]. Among the functional consequences, TTFields significantly downregulated VEGF, HIF-1α, and matrix metalloproteinase (MMP)-2 and MMP-9, resulting in impaired angiogenic capacity in three-dimensional culture models [29]. This anti-angiogenic effect carries direct TME relevance: vascular normalization within the tumor bed reduces hypoxia-driven immunosuppression, decreases TAM recruitment, and may improve drug delivery—independently of and additively to the BBB permeability effects described in Section 7.

**AMPK activation and autophagy.** TTFields-induced mitotic disruption generates aneuploid daughter cells characterized by elevated ER stress, decreased intracellular ATP levels, and proteotoxic stress—a cellular state that converges on activation of AMP-activated protein kinase (AMPK), a master metabolic sensor and regulator of autophagic flux [30]. Shteingauz and colleagues demonstrated that TTFields upregulate autophagy in glioma cells in an AMPK-dependent manner, evidenced by increased LC3-II lipidation, accumulation of LC3 puncta and autophagosome-like structures visualized by fluorescence and transmission electron microscopy, and sequential activation of the AMPK/ULK1 pathway [30]. Critically, this autophagy induction was spatiotemporally coupled to mitotic disruption: time-lapse microscopy revealed that the increase in LC3 puncta formation was specific to cells that had undergone aberrant division during TTFields application, localizing the autophagy response to the product of TTFields-induced mitotic catastrophe rather than representing a general cellular stress response [30].

The functional significance of TTFields-induced autophagy is dualistic and contextually determined. In the Shteingauz model, autophagy operated as a cytoprotective survival mechanism: genetic depletion of AMPK or the autophagy effector ATG7 sensitized glioma cells to TTFields, and pharmacological inhibition of autophagy with chloroquine (CQ) produced a significant dose-dependent enhancement of TTFields-mediated cell death [30]. This survival function of autophagy represents a resistance mechanism that defines a rational therapeutic target—the TTFields + chloroquine or hydroxychloroquine combination—with direct clinical translatability given the established CNS penetration of both agents. However, autophagy also functions as an upstream contributor to immunogenic cell death under conditions of excessive autophagic flux, releasing DAMPs, including ATP and HMGB1, that reinforce the innate immune activation cascade described in Section 4 [19]. Whether TTFields-induced autophagy in GBM cells operates predominantly in the cytoprotective or the immunostimulatory mode is likely determined by the intensity and duration of TTFields exposure, the metabolic state of the target cell, and the concurrent therapeutic context—an unresolved question with important implications for optimizing TTFields-based combination schedules.

Taken together, the intracellular signaling effects of TTFields described in this section collectively converge on the GBM TME through three principal routes: inhibition of invasion limits tumor spread and maintains the anatomical integrity of immune exclusion zones available for targeting; anti-angiogenic effects reduce hypoxia-mediated immunosuppression and improve local immune cell trafficking; and autophagy modulation influences the immunogenicity of dying cells, connecting cytoskeletal and metabolic stress responses to the innate immune activation framework established in the preceding sections. The key preclinical studies supporting TTFields-TME interactions across the biological domains examined in Section 4, Section 5, Section 6, Section 7 and Section 8 are summarized in Table 1.

## 9. TTFields and Radiotherapy: Mechanistic Convergences and Clinical Implementation

### 9.1. Radiobiological Convergences at the DNA Damage Interface

The interaction between TTFields and ionizing radiation at the cellular level is biologically grounded and experimentally validated, extending well beyond the simple additive antiproliferative effects that would be expected from two independent cytotoxic modalities. The early preclinical work by Giladi and colleagues established that TTFields applied after radiotherapy (RT) significantly impair the repair of radiation-induced DNA double-strand breaks (DSBs) in glioma cells [31]. Using the alkaline comet assay and γH2AX foci analysis in U-118 MG and LN-18 cell lines, this study showed that most RT-induced DNA damage was repaired within 24 h in untreated controls. By contrast, more than 40% of the initial damage remained unrepaired when TTFields were applied 1–2 h after irradiation. A comparable effect was observed with bleomycin as an alternative DNA-damaging agent, suggesting a mechanism independent of the specific type of lesion [31]. Importantly, ATM phosphorylation kinetics were not altered by TTFields, indicating that the impairment operates downstream of the initial damage recognition step, likely at the level of repair execution—a finding consistent with the homologous recombination (HR) impairment subsequently demonstrated by Karanam and colleagues, who showed that TTFields downregulate BRCA1 in NSCLC cell lines and thereby generate a conditional HR deficiency that renders tumor cells selectively vulnerable to ionizing radiation, whose cytotoxic effects are disproportionately dependent on HR for the faithful resolution of DSBs in the S and G2 phases of the cell cycle [24]. More recent work indicates that TTFields-induced HR suppression is not confined to BRCA1 but reflects a broader disturbance of the Fanconi anemia–BRCA–RAD51 network. Across several tumor histologies, including ovarian cancer models, TTFields coordinately downregulate core and downstream FA-BRCA components—FANCB, FANCD2, FANCJ and BRCA2—producing an HR-deficient (“BRCAness”) phenotype with attendant synergy toward DNA-damaging agents and PARP inhibitors [25]. Because BRCA2 mediates the loading of RAD51 onto resected DNA ends, its suppression is expected to compromise RAD51-dependent strand invasion, the central catalytic step of HR. The convergence of these mechanisms—HR pathway suppression and persistence of unresolved γH2AX foci, with consequent reduction in clonogenic survival—defines a mechanistic basis for genuine radiosensitization that extends beyond cytostasis. The partner-dependence of this HR vulnerability merits emphasis. Whereas HR resolves the DSBs generated directly by ionizing radiation—the basis of the radiosensitization noted above—in proliferating glioma cells, HR is also the principal pathway repairing the secondary DSBs that arise from temozolomide-induced O6-methylguanine lesions; accordingly, RAD51 or BRCA2 depletion sensitizes glioma cells to temozolomide and other alkylating agents, an effect abolished by MGMT expression and not observed for ionizing radiation alone [32]. TTFields-mediated HR suppression, therefore, constitutes a shared upstream node capable, in principle, of sensitizing GBM cells both to concurrent radiotherapy and to adjuvant temozolomide (Section 6 and Section 10).

Shared mechanisms further amplify this radiobiological synergy at the level of mitotic catastrophe. Both ionizing radiation and TTFields drive tumor cells into aberrant mitosis: RT by inducing premature mitotic entry of cells with unrepaired DNA damage through checkpoint adaptation, and TTFields by directly disrupting spindle assembly and cytokinesis [13,14,31]. Cells that enter mitosis with unrepaired or improperly repaired DSBs—a state exacerbated by the combination—are disproportionately susceptible to mitotic catastrophe, a mode of cell death associated with nuclear envelope rupture, micronucleus formation, and downstream cGAS/STING activation, creating a direct bridge between the radiobiological and immunological mechanisms addressed in the following subsection [7,31].

A practical radiobiological concern—the potential perturbation of the radiation dose distribution by the transducer arrays—was addressed by Giladi and colleagues using a phantom model, and subsequently by Guberina and colleagues in the context of a clinical phase I trial using non-coplanar intensity-modulated RT [31,33]. Both analyses demonstrated that dose deviations within the clinical target volume attributable to the transducer arrays were not clinically significant, supporting the feasibility of concurrent delivery from a dosimetric standpoint [31]. The temporal sequencing used in the Giladi preclinical model—TTFields applied after, rather than simultaneously with, each radiation fraction—is rational, as it allows the radiation to induce the initial DSB load that TTFields then prevents from being repaired, and is technically feasible with the current clinical workflow whereby arrays are removed during RT delivery and reapplied immediately thereafter.

### 9.2. Immunological Synergy: A Double Hit on the GBM Microenvironment

The immunological consequences of combining TTFields with RT in the GBM TME represent the most translationally novel aspect of their interaction and the area of greatest mechanistic convergence with the innate and adaptive immune pathways described in Section 4 and Section 5. Ionizing radiation is itself a well-characterized inducer of immunogenic cell death and cGAS/STING pathway activation: RT-induced DSBs, when incompletely repaired, generate cytoplasmic chromatin fragments and micronuclei that are recognized by cGAS, driving STING-dependent type I IFN production and the downstream DC maturation and T cell priming cascade [7]. The molecular sequence is directly analogous to that induced by TTFields-mediated nuclear envelope rupture, suggesting that the combination of RT and TTFields may deliver a quantitatively amplified and qualitatively sustained “double hit” on innate immune sensing in the GBM TME.

As detailed in Section 4, TTFields activate cGAS/STING signaling and AIM2 inflammasome engagement through nuclear envelope rupture and micronucleus formation; ionizing radiation engages the same innate sensing pathways through an analogous but mechanistically distinct route involving RT-induced DSBs and impaired chromatin repackaging. Several additional mechanistically distinct but temporally convergent immune-modulatory effects further support this hypothesis. First, TTFields and RT both induce nuclear envelope disruption and micronucleus formation through different primary mechanisms—TTFields via cytoskeletal destabilization and aberrant cytokinesis, RT via direct DNA strand breakage and checkpoint bypass—generating overlapping pools of cytosolic dsDNA that simultaneously engage cGAS/STING and AIM2 inflammasome sensing [7,31]. Second, RT upregulates surface MHC-I expression on GBM cells and enhances tumor antigen release, potentiating the antigen cross-presentation capacity of DCs that are concurrently being matured by TTFields-driven type I IFN [6,7]. Third, both RT and TTFields independently upregulate PD-L1 through IFN-γ-dependent JAK/STAT signaling (Section 5). The resulting compounded adaptive immune resistance pressure on tumor cells and infiltrating myeloid populations constitutes the strongest biological rationale yet for a triple combination of TTFields, RT, and PD-1/PD-L1 blockade in newly diagnosed GBM [6,7,19].

**Figure 2:**
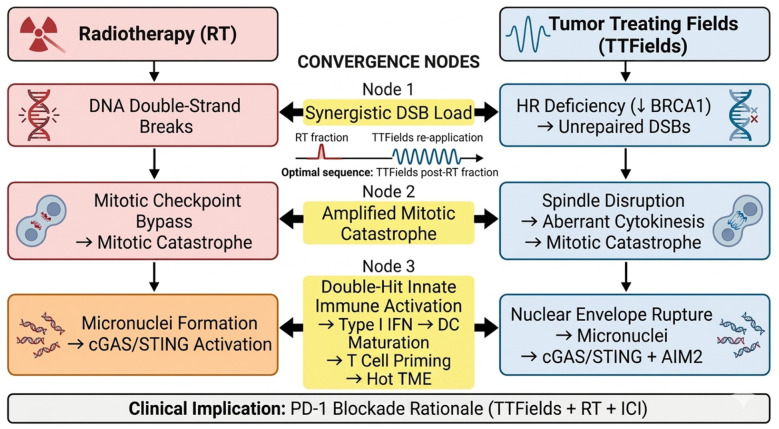
Schematic of the mechanistic convergence between radiotherapy and TTFields in the glioblastoma tumor microenvironment, summarizing the shared nodes of DSB accumulation, HR impairment, micronucleus formation, cGAS/STING and AIM2 inflammasome activation, and downstream adaptive immune priming. RT (**left**, red) and TTFields (**right**, blue) engage three sequential convergence nodes (yellow): synergistic DSB accumulation through RT-induced strand breakage and TTFields-mediated HR impairment (Node 1; inset shows optimal post-fraction TTFields re-application sequence); amplified mitotic catastrophe (Node 2); and double-hit innate immune activation via cGAS/STING and AIM2 inflammasome engagement, driving type I IFN production, DC maturation, and T cell priming toward a “hot” TME (Node 3). The combined mechanistic rationale supports a TTFields + RT + ICI triple combination strategy. Abbreviations: AIM2, absent in melanoma 2; cGAS, cyclic GMP-AMP synthase; DC, dendritic cell; DSB, double-strand break; HR, homologous recombination; ICI, immune checkpoint inhibitor; IFN, interferon; RT, radiotherapy; STING, stimulator of interferon genes; TME, tumor microenvironment; TTFields, Tumor Treating Fields.

The timing of TTFields relative to RT is mechanistically important for the immunological component of their interaction. The cGAS/STING activation induced by TTFields requires cytosolic DNA accumulation secondary to nuclear envelope disruption during aberrant mitosis—a process that unfolds over hours to days of continuous field exposure [7]. RT-induced micronucleus formation similarly peaks in the days following irradiation. Sequential delivery—TTFields initiated immediately after each radiation fraction—may therefore allow the two inducers of cytosolic DNA to operate synergistically within overlapping temporal windows, amplifying the total innate immune stimulus delivered to the TME across the radiotherapy treatment course. Whether truly concurrent delivery during RT fractions (technically precluded by the current practice of array removal during beam-on) would further enhance or potentially saturate the cGAS/STING signaling capacity of tumor cells is an open question that awaits dedicated preclinical modeling. The convergent mechanisms of DNA damage repair impairment, mitotic catastrophe, and innate immune activation shared by RT and TTFields, together with their sequential temporal relationships, are schematically illustrated in Figure 2.

### 9.3. Clinical Implementation: Workflow, Feasibility, and Toxicity Management

The clinical integration of TTFields with the radiotherapy phase of GBM treatment—as opposed to the maintenance phase evaluated in the EF-14 trial—has been evaluated in two prospective feasibility studies that collectively define the current clinical workflow for concurrent delivery.

Bokstein and colleagues conducted the first prospective safety and feasibility study of concurrent TTFields, RT, and TMZ in newly diagnosed GBM, enrolling ten patients who received standard chemoradiation (60 Gy in 30 fractions, daily TMZ 75 mg/m^2^) with TTFields at 200 kHz applied continuously, with arrays removed only during each radiation fraction and reapplied immediately thereafter [34]. The concurrent regimen was feasible and safe, with acceptable compliance rates and no unexpected treatment-related toxicities beyond the established profile of each individual modality [34]. Dermatological adverse events—the primary toxicity of TTFields—were predominantly grade 1–2 skin reactions at array contact sites, manageable with standard topical corticosteroids and optimized array positioning protocols [34].

The central practical challenge of concurrent TTFields-RT delivery is the spatial conflict between transducer array placement on the scalp and the dose distribution required to treat the tumor bed, particularly regarding scalp-sparing dose constraints that are increasingly incorporated into modern GBM radiotherapy planning. The SPARE approach, developed by Miller, Song, and colleagues, addresses this challenge through a scalp-sparing radiation technique (the SPARE trial) that integrates array positioning maps into the treatment planning workflow, optimizing array layout to minimize overlap between high-dose scalp regions and transducer contact areas. In the published SPARE series, this approach significantly reduced the incidence and severity of scalp reactions compared to conventional array placement without scalp-sparing optimization, maintaining device compliance and enabling prolonged concurrent use [35]. The registrational-intent evidence base for concurrent TTFields-RT is being established by the TRIDENT trial (EF-32; NCT04471844), an international phase 3 randomized study comparing the triple combination of TTFields (200 kHz) concomitant with standard chemoradiation (RT/TMZ) followed by maintenance TTFields/TMZ, versus standard RT/TMZ followed by maintenance TTFields/TMZ in all patients [12]. Critically, the TRIDENT design addresses the central unanswered question of whether initiating TTFields during the radiotherapy phase—rather than after its completion as in EF-14—translates the mechanistic synergies described in Section 9.1 and Section 9.2 into a survival benefit. The trial has completed enrollment of 981 patients, powered for an OS hazard ratio of less than 0.8, and its results are awaited as the definitive prospective test of the TTFields-RT combination hypothesis [12]. An overview of the principal clinical studies evaluating TTFields-based combination strategies in GBM, together with their principal methodological limitations, is provided in Table 2. Except for the maintenance-setting EF-14 trial, the current clinical evidence for TTFields combinations—and, in particular, for concurrent delivery with radiotherapy—rests predominantly on small, single-arm feasibility, dosimetric, and cohort studies that are not powered for efficacy. Level 1 evidence for the concurrent and immunotherapy-based strategies awaits the ongoing phase 3 trials (TRIDENT and EF-41/KEYNOTE-D58).

**Figure 3:**
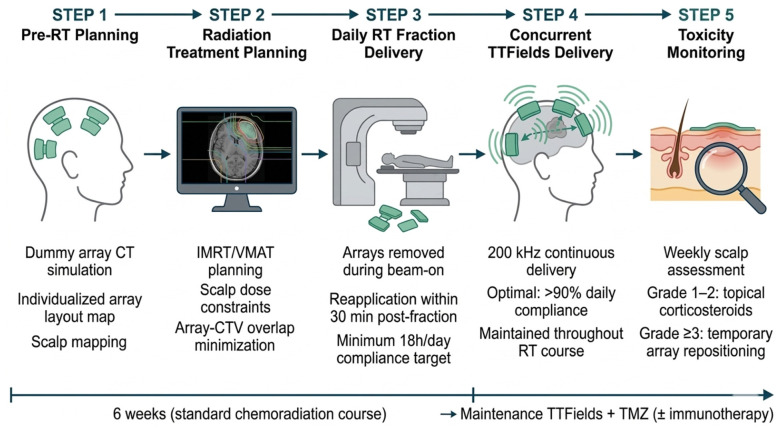
Clinical workflow for concurrent TTFields and radiotherapy in newly diagnosed glioblastoma. Sequential steps include dummy array CT simulation with individualized scalp mapping (Step 1), IMRT/VMAT planning with scalp dose constraints and array-CTV overlap minimization (Step 2), array removal during beam-on with reapplication within 30 min post-fraction (Step 3), continuous 200 kHz TTFields delivery targeting ≥90% daily compliance throughout the six-week chemoradiation course (Step 4), and weekly scalp toxicity assessment with topical management (Step 5). Abbreviations: CTV, clinical target volume; IMRT, intensity-modulated radiotherapy; RT, radiotherapy; TMZ, temozolomide; TTFields, Tumor Treating Fields; VMAT, volumetric modulated arc therapy.

From a radiation oncology workflow perspective, the practical implementation of concurrent TTFields-RT requires several adaptations. CT simulation should be performed with dummy transducer arrays in situ to accurately characterize the dosimetric impact of the arrays and to generate an individualized array layout map that is incorporated into the treatment plan. Non-coplanar IMRT or volumetric modulated arc therapy (VMAT) planning should account for array-induced dose perturbations, which—while dosimetrically modest—can affect superficial scalp doses in the regions of array contact [33]. Array removal immediately before each radiation fraction and reapplication within 15–30 min of fraction completion maintains both compliance and the mechanistic post-irradiation TTFields effect on DNA repair described in Section 9.1. Scalp assessment at weekly on-treatment visits should include inspection of array contact sites, with topical management initiated at first sign of grade 1 reaction to prevent progression to grade 2–3 skin breakdown that would necessitate treatment interruption. The proposed clinical workflow for concurrent TTFields-RT delivery—as evaluated in published feasibility studies—from CT simulation to toxicity management, is summarized in Figure 3.

### 9.4. Open Questions and Research Priorities

Despite the mechanistic rationale and initial clinical feasibility data, several fundamental questions regarding the TTFields-RT interaction remain unanswered and define the research agenda for this combination.

The optimal timing of TTFields initiation relative to RT is the most clinically consequential open question. Current standard practice initiates TTFields after completion of chemoradiation; the emerging concurrent approach described above represents an intensification that is rational but requires validation in adequately powered randomized trials. Whether concurrent initiation during RT improves outcomes over standard post-RT initiation—and for which patient subgroups—is the primary endpoint that the ongoing registrational-intent trial infrastructure should address.

The role of TTFields in hypofractionated and stereotactic radiosurgery (SRS/SBRT) settings remains unexplored. Hypofractionated regimens (e.g., 40 Gy in 15 fractions for elderly or MGMT-unmethylated patients) and focal re-irradiation at recurrence represent clinical contexts in which the biological interactions between TTFields and RT may differ substantially from those observed with standard fractionation, given the different mechanisms of cell killing, the altered immune responses associated with high-dose-per-fraction RT, and the distinct tumor cell populations targeted. Whether the cGAS/STING amplification described for conventionally fractionated RT also operates—or is potentially enhanced—with hypofractionated or ablative dose regimens is both a mechanistic question of immediate interest and a clinical question of growing practical relevance, given the increasing use of hypofractionation in GBM management.

The re-irradiation setting at tumor recurrence represents a particularly underexplored context for TTFields-RT combination, where the mechanistic rationale for immune sensitization may be especially relevant given the post-chemoradiation immune suppression and the limited efficacy of available salvage options.

Finally, the identification of predictive biomarkers for TTFields-RT synergy—at the genomic (MGMT methylation, HR pathway mutations), transcriptomic (cGAS/STING expression, IFN signature), or imaging (perfusion MRI BBB permeability parameters) levels—is a prerequisite for the rational, patient-selection-driven design of future combination trials.

## 10. Therapeutic Combinations Beyond Radiotherapy: A TME-Centric Perspective

The breadth of TTFields effects on the GBM TME described in the preceding sections defines a biological landscape that is intrinsically combinatorial: each mechanism—innate immune activation, adaptive T cell priming, GSC targeting, BBB modulation, DDR impairment, and metabolic reprogramming—identifies a corresponding therapeutic vulnerability that can be pharmacologically exploited. This section does not aim to provide a comprehensive catalog of TTFields combination strategies, which has been addressed elsewhere [3,4], but rather to organize the most clinically proximate combinations according to the TME mechanisms they engage, as a framework for rational trial design.

**TTFields and temozolomide.** The established TTFields-TMZ maintenance combination exerts complementary effects on the GBM TME beyond direct tumor cell killing. TMZ-induced lymphodepletion—a well-recognized on-target immunological liability of alkylating chemotherapy—may paradoxically generate a homeostatic lymphocyte rebound, a phenomenon described in other tumor systems, which could potentially be exploited to amplify adoptive or vaccine-based immune strategies in appropriately selected patients. The opposing immunological effects of TMZ (immunosuppressive at conventional doses) and TTFields (immunostimulatory via cGAS/STING activation and ICD) within the same treatment window represent an undercharacterized pharmacodynamic interaction with potential consequences for the immune contexture of the TME that merit systematic characterization [7,19]. Beyond these immunological considerations, the combination has a distinct DNA-repair rationale. The cytotoxicity of TMZ derives chiefly from O6-methylguanine lesions that, after futile mismatch repair, are converted into replication-associated DSBs whose resolution in proliferating glioma cells depends on homologous recombination; experimental depletion of RAD51 or BRCA2 markedly sensitizes glioma cells to TMZ, an effect confined to MGMT-deficient cells and not seen with ionizing radiation [32]. Because TTFields suppress the FA-BRCA-RAD51 network and impose a BRCAness-like state across tumor types [25], they may act as a homologous-recombination-directed sensitizer to alkylating chemotherapy. This interaction is of particular interest for the adjuvant TMZ phase that follows concurrent RT-TMZ, where TTFields are already delivered as maintenance and could, in principle, lower the HR-dependent threshold for TMZ-induced cell death in residual proliferating tumor cells that are insufficiently protected by MGMT. Because the homologous-recombination-dependent component of TMZ cytotoxicity is engaged only once O6-methylguanine lesions escape MGMT-mediated repair, this mechanism is expected to operate in MGMT-deficient (promoter-methylated) cells; whether TTFields-mediated HR suppression can also overcome the MGMT-proficient resistance that characterizes MGMT-unmethylated tumors remains unproven and warrants specific testing.

**TTFields and immune checkpoint inhibitors.** The mechanistic and clinical rationale for combining TTFields with PD-1/PD-L1 blockade has been developed in detail in Section 5 and is the most advanced combination in clinical translation, anchored by the 2-THE-TOP phase 2 data and the registrational-intent EF-41/KEYNOTE-D58 trial [9,21]. The key open question from a TME perspective is whether the addition of RT to this doublet—as is now being evaluated in emerging triple combination designs—creates a cGAS/STING amplification sufficient to meaningfully overcome the multiple non-PD-1-mediated immunosuppressive mechanisms that have thus far limited ICI efficacy in GBM [6,7].

**TTFields and autophagy inhibition.** The AMPK-dependent cytoprotective autophagy induced by TTFields in glioma cells, characterized in Section 8, defines an experimentally validated resistance mechanism with a directly actionable pharmacological target [30]. Chloroquine and hydroxychloroquine—both with established CNS penetration and known safety profiles in GBM patients—inhibit autophagic flux at the lysosomal level and demonstrate dose-dependent synergy with TTFields in vitro [30]. This combination warrants prospective clinical evaluation, particularly given the accessibility of both agents and the absence of overlapping toxicities with standard chemoradiation.

**TTFields and DNA damage response inhibitors.** The HR impairment induced by TTFields in both bulk GBM cells and patient-derived GSC models [24,31], combined with direct sensitization of GSCs to PARP and ATR inhibitors demonstrated by Vanderlinden and colleagues [10], provides a mechanistic framework for TTFields-DDRi combinations that is analogous to the synthetic lethality exploited in BRCA-mutated cancers and may be particularly relevant in the MGMT-unmethylated GBM subgroup, where TMZ efficacy is limited, and alternative cytotoxic strategies are most urgently needed.

**TTFields and drug repurposing.** The CUSP9v3 strategy—a nine-drug repurposing cocktail combined with metronomic TMZ—demonstrated synergistic anti-GBM activity when combined with TTFields in vitro across established cell lines, primary cultures, and stem-like GBM cells, with evidence of metabolic reprogramming including marked reduction in oxidative phosphorylation and mitochondrial respiratory chain complex expression [36]. The favorable safety profile of CUSP9v3 documented in a phase Ib/IIa trial facilitates clinical translation of this multimodal approach, and the metabolic vulnerability it exploits is functionally complementary to the DDR and cytoskeletal disruption exerted by TTFields.

## 11. Limitations of the Current Evidence Base

The mechanistic framework assembled above rests on an evidence base with several structural limitations. These are considered below along six axes.

Translational limitations of the preclinical evidence. Much data on TTFields–TME interaction derive from in vitro systems using established cell lines or short-term patient-derived cultures, which do not recapitulate the three-dimensional architecture, cellular heterogeneity, and immune privilege of the human GBM TME. The syngeneic orthotopic models underlying the most informative immunological studies [7,19] employ non-human tumor cells in immune backgrounds that differ substantially from the lymphopenic, corticosteroid-exposed state of patients undergoing concurrent chemoradiation. Moreover, in vitro field delivery is applied under geometrically simplified, temperature-controlled conditions that do not replicate the heterogeneous field distribution produced by transducer arrays on an anatomically complex head, with variable skull geometry, scalp conductivity, and tumor depth [13,15]. These discrepancies limit the direct translatability of preclinical mechanistic findings and define a priority gap for computational and ex vivo modeling.

Uncertain magnitude of immune activation in human GBM. The GBM TME is not a single-layer immunosuppressive barrier but a redundant, multi-mechanism ecosystem in which even robust innate immune activation may be sequentially extinguished by Treg expansion, MDSC-mediated arginine depletion, TIM-3/LAG-3 co-inhibitory upregulation, and IDO1-driven tryptophan catabolism [5,6]. Whether the degree of TME reprogramming achievable with TTFields, alone or with RT, is quantitatively sufficient to shift the TME from cold to immunologically active in most patients—or only in favorable subsets—remains unresolved. Critically, the available human immune-correlate data are confined to a small single-arm correlative cohort in which activation was inferred largely from peripheral blood rather than serial intratumoral profiling [9].

Corticosteroid-associated immunosuppression. Most GBM patients receive dexamethasone for peritumoral edema during and after chemoradiation. Glucocorticoids are profoundly lympholytic and suppress T-cell activation and dendritic-cell function, and baseline corticosteroid exposure has been linked to attenuated responses to immunotherapy in neuro-oncology [6]. Any TTFields-driven adaptive immune activation is therefore likely to be blunted in steroid-exposed patients, yet none of the human immune-correlate datasets generated to date—including those from the 2-THE-TOP trial—have been systematically stratified by concurrent corticosteroid dose. The interaction between steroid exposure, baseline lymphopenia, and the magnitude of TTFields-induced immune priming is largely uncharacterized and constitutes a major confounder for immune-integrated combination strategies. We therefore recommend that future immune-endpoint trials of TTFields incorporate concurrent corticosteroid dose as a pre-specified covariate—and, where feasible, as a stratification factor or an exclusion criterion for immune-correlate sub-studies—so that TTFields-attributable immune effects can be separated from steroid-driven lymphodepletion.

Blood–brain barrier variability. The evidence for TTFields-mediated BBB modulation derives from rodent and in vitro models and from small human imaging series [11,26,27], and its clinical exploitation is constrained by several sources of variability. BBB integrity is markedly heterogeneous within a single tumor—partially disrupted in the enhancing core but largely preserved in the infiltrative margin from which recurrence arises—so that any permeability gain may be unevenly distributed across the compartments that matter most. The frequency optimum for barrier opening (100 kHz) differs from that approved for anti-mitotic activity (200 kHz), and whether the permeability changes observed at 200 kHz are sufficient to raise parenchymal concentrations of co-administered agents has not been demonstrated in prospective pharmacokinetic studies. Inter-patient differences in scalp and skull geometry, tumor depth, and array layout further modulate the delivered field and, presumably, the magnitude of any BBB effect.

Compliance-related bias in efficacy estimates. The frequently cited association between device compliance and survival—a median overall survival of 24.9 months among patients with >90% compliance in the EF-14 subgroup analysis [17]—warrants methodological caution. This was a post hoc, non-randomized subgroup comparison, and compliance is not an independent intervention, but a behavior strongly correlated with favorable prognostic factors, including better performance status, slower neurological deterioration, greater social support, and absence of early progression. Because classification as highly compliant over a prolonged period also requires surviving that period, compliance-stratified survival conflates a plausible field-exposure dose–response with healthy-adherer and guarantee-time (immortal-time) biases. While a genuine dose–response is biologically reasonable and consistent with the conceptual analogy to radiotherapy fractionation, the apparent magnitude of benefit in high-compliance subgroups should not be interpreted as a direct causal estimate of the TTFields effect. Future trials could mitigate these biases by treating compliance as a time-varying covariate rather than a fixed baseline attribute, by applying landmark analyses or marginal structural models that condition on survival to a defined initial timepoint, and—most definitively—by randomizing prescribed field-exposure targets rather than inferring a dose–response from observed adherence.

Unresolved sequencing and optimization with radiotherapy and immunotherapy. The optimal temporal integration of TTFields with radiotherapy (concurrent versus post-chemoradiation initiation), with immune checkpoint blockade, and within hypofractionated or re-irradiation regimens remains undefined. These questions are well-grounded but experimentally open and are developed as forward-looking challenges in Section 12. Their resolution is compounded by the current absence of validated predictive biomarkers, since combination trials have largely proceeded without a means of selecting patients most likely to benefit from the specific TME-modulatory mechanism being engaged.

## 12. Current Controversies and Future Challenges

### 12.1. Current Controversies

Several aspects of TTFields biology and clinical application remain genuinely contested, and a balanced appraisal must engage with them explicitly.

**The interpretive weight of an unblinded pivotal trial.** The clinical rationale for TTFields ultimately derives from the EF-14 trial, which was conducted open-label, with control patients not wearing an inactive (sham) device [2]. This design has generated ongoing debate. Skeptics argue that the survival advantage could be inflated by the more intensive clinical contact inherent to device use, by performance or placebo effects, and by the compliance-related selection discussed in Section 11; proponents counter that overall survival is a hard endpoint relatively resistant to placebo influence, that the effect size was substantial and internally consistent, and that requiring continuous sham-device wear for the duration of the disease course raises ethical and practical objections.

**Immunostimulation versus immunosuppression: a contested net effect.** In keeping with the prevailing literature, this review emphasizes the pro-immunogenic actions of TTFields—cGAS/STING engagement, immunogenic cell death, and M2-to-M1 repolarization. Whereas Section 11 questioned whether the magnitude of this activation is sufficient to remodel the human GBM TME, a distinct and more fundamental uncertainty concerns its direction, since the same pathways carry a well-documented capacity for the opposite effect. Sustained STING activation can promote T-cell exhaustion and tolerogenic programs; type I interferon signaling is biphasic and context-dependent; and TTFields-induced autophagy is, in several models, cytoprotective rather than immunogenic [7,30]. Whether the net immunological consequence of prolonged TTFields exposure in the human GBM TME is a shift toward productive antitumor immunity, or whether, in some patients, it could paradoxically reinforce immunosuppression, is not established and cannot be resolved from current preclinical data. Clarifying the directionality and durability of this effect—ideally through serial intratumoral profiling rather than peripheral surrogates—is arguably the single most important mechanistic question for the field.

**Frequency optimization and the BBB trade-off.** A specific technical controversy, introduced as a limitation in Section 11, concerns the divergence between the frequency that maximizes BBB permeabilization (≈100 kHz) and that approved for anti-mitotic efficacy in GBM (200 kHz) [26,27]. Whether the BBB-modulatory effect demonstrable at 200 kHz is of sufficient magnitude to be therapeutically useful for drug delivery—or whether realizing this benefit would require frequency modulation that compromises cytotoxic efficacy—remains disputed and unresolved by available human data.

**Patient burden, quality of life, and cost.** Finally, a clinically consequential debate concerns the balance between benefit and burden. The formal health-related quality-of-life analysis of EF-14 was largely reassuring, showing no significant net deterioration across most HRQoL domains with the addition of TTFields, aside from more frequent scalp itch related to the transducer arrays [37]. Nonetheless, continuous wear of scalp arrays for ≥18 h per day over months to years imposes a real adherence and psychosocial demand, predictable dermatological toxicity, and considerable financial cost. The cost-effectiveness of TTFields is itself contested: European analyses have reported incremental cost-effectiveness ratios well above conventional willingness-to-pay thresholds, attributing this primarily to device cost [38,39], whereas a US payer–perspective analysis judged the addition of TTFields cost-effective relative to contemporary oncology benchmarks (ICER ≈ USD197,000 per quality-adjusted life-year) [40]. The degree to which the survival benefit justifies this burden—and for which patients—therefore remains debated, with direct implications for equitable access. Compounding this, robust real-world effectiveness data—registry-based survival, adherence, and toxicity outside the controlled trial setting—remain limited, which further constrains the generalizability of the pivotal-trial estimates to routine practice.

### 12.2. Future Challenges and Research Priorities

The controversies above, together with the limitations enumerated in Section 11, define a research agenda that extends beyond the radiotherapy-specific priorities of Section 9.4. Three cross-cutting challenges merit emphasis.

**From additive testing to mechanism-guided, biomarker-stratified design.** The dominant challenge is conceptual: combination strategies have largely been tested additively, without selecting patients by the TME mechanism being engaged. The field requires a shift toward biomarker-stratified, immune-integrated protocols in which candidate predictive markers are collected prospectively rather than retrospectively. The most clinically actionable unresolved questions, their translational implications, candidate biomarkers, and proposed study designs are summarized in Table 3.

**Predictive biomarkers and emerging profiling technologies.** Rational patient selection for TTFields-containing strategies will ultimately depend on predictive biomarkers that are, at present, largely aspirational. Genomic candidates include MGMT promoter methylation—already prognostic and predictive for temozolomide—and markers of homologous-recombination status (BRCA/FA-pathway expression or functional HR assays), which the radiosensitization and temozolomide-sensitization data discussed above nominate as candidate predictors of TTFields–DNA-damage synergy [24,25,32]. Transcriptomic candidates include tumor cGAS/STING and type I interferon signatures, whose strength correlated with T-cell-receptor clonal expansion in the only available human dataset [9], alongside immune-cell infiltration patterns and tumor mutational burden. Because no single marker is likely to capture a process as distributed as microenvironmental remodeling, a logical direction is the development of composite immune scores integrating interferon-response, T-cell-infiltration, macrophage-polarization, and STING-pathway activity into a single index—analogous to the single-sample GSEA (ssGSEA)-derived immune prognostic models recently reported in glioblastoma, in which an immunosuppressive chemokine program involving tumor-associated microglia and macrophages stratified overall survival across independent cohorts [41]. More broadly, rational immune-integrated patient selection will need to consider an immune-target and signaling landscape extending beyond the cGAS/STING axis emphasized here, encompassing additional co-inhibitory checkpoints and JAK/STAT-type signaling nodes [42]. Realizing such biomarkers will require profiling technologies suited to dynamic, multicellular interactions: single-cell and spatial transcriptomics to resolve TTFields-induced shifts across tumor, immune, and stromal compartments at the invasive margin [18], and minimally invasive monitoring through liquid biopsy. For the latter, cerebrospinal fluid cell-free DNA and methylation profiling are likely more informative than plasma in GBM, where preserved barrier compartments limit plasma cell-free DNA shedding, and could in principle offer a non-invasive readout of treatment-induced microenvironmental change. The broader development of pan-cancer cfDNA methylation panels [43] supports the analytical feasibility of this approach, although its adaptation to cerebrospinal-fluid compartments in GBM remains to be established. These approaches are proposed here as a research agenda rather than as validated tools.

**Combinatorial in situ vaccination.** The combination of TTFields with oncolytic virotherapy, which similarly exploits immunogenic cell death for immune priming, offers a conceptually attractive “dual in situ vaccination” strategy in which the two modalities may engage complementary, non-redundant innate sensing pathways; this warrants preclinical evaluation in orthotopic GBM models before clinical translation.

**Cellular immunotherapy and the recurrent setting.** Whether TTFields-mediated BBB modulation and TAM repolarization can enhance the intratumoral trafficking of adoptively transferred cells (e.g., CAR-T) is an open, high-value question (Table 3). The re-irradiation setting at recurrence—where TTFields is among the few salvage options with an established safety profile, and where the immunological contexture has been reshaped by prior chemoradiation—likewise remains an underexplored but clinically urgent context for combination evaluation (Section 9.4).

## 13. Conclusions

Tumor Treating Fields have entered the clinical management of GBM as a regulatory-approved component of standard-of-care therapy in multiple jurisdictions, yet their integration into oncological practice has proceeded largely on the basis of survival data from the EF-14 trial rather than on a mechanistic understanding of how they interact with the tumor’s biological ecosystem. The evidence reviewed here supports a substantial reconceptualization: TTFields are not merely an anti-mitotic device that acts in parallel with other treatment modalities, but a pleiotropic modulator of the GBM TME whose effects on innate immune sensing, adaptive T cell priming, GSC biology, BBB permeability, intracellular signaling, and DNA damage repair position it as a candidate biological platform that may enhance the efficacy of immunological, cytotoxic, and radiotherapeutic strategies—a possibility that remains to be confirmed in prospective clinical studies.

From a radiation oncology perspective, the convergence between TTFields and RT at the level of the TME is particularly consequential. Both modalities independently activate cGAS/STING-dependent innate immunity through nuclear envelope disruption and cytosolic DNA accumulation, impair homologous recombination-mediated DNA repair, drive immunogenic cell death, and upregulate PD-L1—creating a strong mechanistic rationale for therapeutic synergy that extends beyond radiosensitization to encompass TME immunological reprogramming—a hypothesis that awaits prospective validation in randomized trials. The feasibility of concurrent delivery has been established in prospective studies, scalp-sparing techniques have mitigated the principal dermatological barrier to compliance, and the translational trajectory from the 2-THE-TOP phase 2 data to the EF-41/KEYNOTE-D58 registrational trial demonstrates that the field is moving toward immune-integrated combination strategies with appropriate mechanistic grounding.

What is now required is a deliberate shift in trial design philosophy: from sequential, additive combination testing toward mechanistically informed, biomarker-stratified, immune-integrated protocols in which TTFields is conceived as the TME-remodeling backbone of a multimodal strategy rather than an adjunct to existing regimens. The open questions delineated in this review—optimal TTFields-RT timing, hypofractionation interactions, predictive immune biomarkers, and the re-irradiation setting—define a tractable and clinically urgent research agenda. Answering them rigorously, with the methodological standards appropriate to the complexity of the GBM immune landscape, represents both the scientific obligation and the therapeutic opportunity of the next decade in neuro-oncology.

## Figures and Tables

**Table 1:** Key preclinical studies investigating the interaction between Tumor Treating Fields (TTFields) and the glioblastoma tumor microenvironment, organized by biological target.

Authors (Year)	Model	TTFields Parameters	TME Component/Mechanism	Key Finding	Ref
**INNATE AND ADAPTIVE IMMUNITY**					
Chen et al. (2022)	Patient-derived GBM lines; syngeneic orthotopic C57BL/6 mouse	200 kHz, 1.0–1.5 V/cm	cGAS/STING; AIM2 inflammasome; innate–adaptive axis	Nuclear envelope rupture → cytosolic dsDNA → dual cGAS/STING + AIM2 activation → type I IFN, IL-1β, pyroptosis, DC maturation, CD8^+^ infiltration; survival benefit abolished in immunodeficient hosts; T cell clonal expansion (scRNA-seq)	[7]
Voloshin et al. (2020)	Multiple cancer lines (NSCLC, ovarian); syngeneic in vivo	150–200 kHz	Immunogenic cell death; PD-1/PD-L1 axis	HMGB1/ATP release, calreticulin exposure (ICD hallmarks); DC engulfment/maturation; TTFields + anti-PD-1 superior to either alone	[19]
Kan et al. (2025)	NSCLC lines; tumor–macrophage co-culture	150 kHz	TAM polarization	M2 → M1 repolarization via GEF-H1/NF-κB/MyD88; ↑ iNOS, TNF-α, IL-12	[20]
**GLIOMA STEM CELLS**					
Silginer et al. (2017)	Patient-derived glioma-initiating cells; GBM lines	200 kHz	GSC viability/motility; autophagy/necroptosis	Reduced GSC viability and migration; non-apoptotic death (autophagy, necroptosis); caspase-independent	[22]
Vanderlinden et al. (2023)	Patient-derived GSC biobank (>110 models, multi-region)	200 kHz	GSC DDR; HR deficiency	Synergy with PARPi (olaparib, niraparib) and ATRi; TTFields impair HR → conditional DDR vulnerability in therapy-resistant GSCs	[10]
Deleyrolle et al. (2023)	GBM lines; patient-derived cultures	200 kHz	Primary cilia; SHH/GLI signaling	TTFields disrupt cilia integrity; sensitization to TMZ; counteract TMZ-induced ciliogenesis	[23]
**BLOOD–BRAIN BARRIER**					
Salvador et al. (2022)	Murine cerebEND in vitro; healthy rat brain in vivo	100 kHz	BBB tight junctions; claudin-5; ROCK	65% TEER reduction; claudin-5/ZO-1 delocalization (ROCK-mediated); gadolinium accumulation (DCE-MRI); enhanced paclitaxel delivery; reversible ≤ 96 h	[26]
Salvador et al. (2023)	Human 3D BBB model (primary HBMVEC + pericytes)	100 kHz	BBB integrity (human model)	Increased paracellular permeability in human system; tight junction delocalization; recovery at 48 h	[11]
**INTRACELLULAR SIGNALING**					
Kim et al. (2016)	GBM lines (U87, U251)	200 kHz	NF-κB; EMT; angiogenesis	↓ NF-κB, MAPK, PI3K/AKT; ↓ VEGF, HIF-1α, MMP-2/9, vimentin; ↑ E-cadherin; impaired 3D angiogenesis	[29]
Voloshin et al. (2020)	GBM lines	200 kHz	Cytoskeleton; focal adhesion; RhoA/ROCK	GEF-H1/RhoA/ROCK activation; disrupted focal adhesion turnover; impaired migration	[28]
Shteingauz et al. (2018)	Glioma cell lines	200 kHz	AMPK; autophagic flux	AMPK/ULK1-dependent LC3-II lipidation; autophagy coupled to mitotic catastrophe; cytoprotective → chloroquine synergy	[30]
**TTFields–RADIOTHERAPY INTERACTION**					
Giladi et al. (2017)	GBM lines (U-118 MG, LN-18)	200 kHz; post-RT	DNA damage repair; γH2AX; HR	>40% unrepaired DSBs at 24 h vs. RT alone; impaired HR; ATM kinetics unchanged; clinically non-significant array dosimetry	[31]
Karanam et al. (2017)	NSCLC cell lines	200 kHz; with RT	BRCA1; HR deficiency; radiosensitization	TTFields downregulate BRCA1 → conditional HR deficiency → enhanced radiation-induced DSB lethality	[24]

**Abbreviations:** ATRi, ATR inhibitor; BBB, blood–brain barrier; DC, dendritic cell; DCE-MRI, dynamic contrast-enhanced MRI; DDR, DNA damage response; dsDNA, double-stranded DNA; DSB, double-strand break; EMT, epithelial-to-mesenchymal transition; GBM, glioblastoma; GSC, glioma stem cell; HBMVEC, human brain microvascular endothelial cells; HIF-1α, hypoxia-inducible factor 1-alpha; HR, homologous recombination; ICD, immunogenic cell death; iNOS, inducible nitric oxide synthase; MMP, matrix metalloproteinase; NSCLC, non-small cell lung cancer; PARPi, PARP inhibitor; ROCK, Rho-associated kinase; scRNA-seq, single-cell RNA sequencing; SHH, Sonic Hedgehog; TAM, tumor-associated macrophage; TEER, transendothelial electrical resistance; TME, tumor microenvironment; TMZ, temozolomide; TTFields, Tumor Treating Fields; VEGF, vascular endothelial growth factor; ZO-1, zonula occludens-1. →, leads to. Bold rows indicate category subheadings.

**Table 2:** Clinical studies evaluating TTFields-based combination strategies in glioblastoma, with study design, patient population, outcomes, and principal limitations.

Trial/Study (*n*)	Phase and Setting	Combination	Primary Endpoint	Key Result	Limitations/Caveats
EF-14 (Stupp et al., 2017 [2]); *n* = 695	Phase III RCT; ndGBM, maintenance post-CRT	TTFields (200 kHz) + TMZ vs. TMZ	PFS (primary); OS (key secondary)	mOS 20.9 vs. 16.0 mo; HR 0.63 (95% CI 0.53–0.76); *p* < 0.001	Open-label; comparator arm received no sham device (potential performance/placebo bias); the >90%–compliance mOS of 24.9 mo is a post hoc, non-randomized subgroup subject to healthy-adherer and guarantee-time bias
TRIDENT/EF-32 (Shi et al. 2023) [12]; *n* = 981 (enrolled)	Phase III RCT; ndGBM, concurrent CRT + maintenance	TTFields concomitant RT/TMZ → maintenance TTFields/TMZ vs. RT/TMZ → maintenance TTFields/TMZ	OS	Enrollment completed; results pending (NCT04471844)	Results not yet available; open-label; will provide the first RCT-level test of concurrent initiation
Bokstein et al. (2020) [34]; *n* = 10	Prospective phase I (safety/feasibility); ndGBM, concurrent CRT	TTFields + RT (60 Gy/30 fr) + concomitant TMZ	Safety/feasibility	Concurrent delivery feasible; toxicity limited to G1–2 scalp reactions	Very small, single arm; not powered for efficacy; feasibility/safety signal only
Guberina et al. (2020) [33]; *n* = 7	Phase I, dosimetric; ndGBM, concurrent CRT	TTFields + non-coplanar IMRT + TMZ	Dosimetric safety	No clinically significant dose deviation within CTV from arrays	Dosimetric endpoint only; small phase I; no clinical efficacy data
SPARE (Miller et al., 2022) [35]; *n* = 30	Prospective cohort; ndGBM, concurrent CRT	Scalp-sparing RT + TTFields + TMZ	Scalp toxicity; compliance	Reduced scalp-reaction severity; compliance maintained without target compromise	Non-randomized, single-institution cohort; surrogate (toxicity/compliance) endpoints; no efficacy comparator
2-THE-TOP (Tran et al., 2025) [9]; *n* = 31 (12 evaluable for pre-pembrolizumab immune analysis)	Phase II, single arm; ndGBM, maintenance post-CRT	TTFields + TMZ + pembrolizumab	Efficacy; immune correlates	Type I IFN T-cell activation in 11/12 pts; cGAS/STING-dependent TCR clonal expansion (r = −0.80, *p* = 0.014); residual burden predictive	Single arm; small evaluable sample; efficacy benchmarked to historical controls; immune effects inferred mainly from peripheral blood; TTFields contribution not separable from TMZ/pembrolizumab
EF-41/KEYNOTE-D58; *n* = 741 (estimated)	Phase III RCT, double-blind, placebo-controlled; ndGBM, maintenance post-CRT	TTFields + TMZ + pembrolizumab vs. + placebo	OS	Active, recruiting (NCT06556563)	No results yet; maintenance setting will not isolate the RT–TTFields interaction

**Abbreviations:** CI, confidence interval; CRT, chemoradiotherapy; CTV, clinical target volume; GBM, glioblastoma; HR, hazard ratio; IFN, interferon; IMRT, intensity-modulated radiotherapy; mOS, median overall survival; ndGBM, newly diagnosed glioblastoma; OS, overall survival; PFS, progression-free survival; RCT, randomized controlled trial; RT, radiotherapy; TCR, T cell receptor; TMZ, temozolomide; TTFields, Tumor Treating Fields. →, leads to/followed by.

**Table 3:** Unresolved questions in TTFields–tumor microenvironment research and their translational implications for clinical trial design.

Biological Question	Clinical Implication	Candidate Biomarker	Ideal Study Design
Does concurrent TTFields during RT amplify cGAS/STING activation relative to post-RT initiation?	Optimal timing of TTFields initiation (concurrent vs. post-CRT)	Tumor type I IFN signature; serial TCR clonality (liquid biopsy)	Randomized phase II: concurrent vs. sequential TTFields initiation; co-primary endpoints OS and immune TME profiling at re-operation
Does HR deficiency predict TTFields-mediated radiosensitization in GBM?	Patient selection for TTFields + RT ± PARPi combinations	BRCA1 expression; HR mutational signature; functional HR assay on tumor tissue	Biomarker-stratified phase II: TTFields + RT ± olaparib in HR-deficient vs. HR-proficient ndGBM
Do TTFields increase BBB permeability sufficiently to enhance CNS drug concentrations?	Rational basis for TTFields + BBB-restricted agents (targeted therapy, ADCs, immunotherapy)	Perfusion MRI Ktrans; DCE-MRI permeability parameters	Pharmacokinetic substudy within existing TTFields trials; CSF drug concentration sampling
Does autophagy inhibition enhance TTFields efficacy in GBM?	Clinical development of TTFields + hydroxychloroquine combination	LC3-II expression; AMPK activation in tumor tissue	Randomized phase II: TTFields + TMZ ± hydroxychloroquine in ndGBM; correlative autophagy biomarker substudy
Does hypofractionated RT + TTFields produce differential immune activation vs. standard fractionation?	Use of TTFields in elderly/poor performance status patients receiving hypofractionated RT	Tumor cGAS/STING expression; IFN-γ signature; NK and T cell infiltration at re-operation	Translational substudy within hypofractionation trials (e.g., 40 Gy/15 fr cohort); serial immune profiling
Can TTFields enhance CAR-T cell intratumoral trafficking through BBB modulation and TAM repolarization?	TTFields as a platform to enable CNS cellular immunotherapy	Intratumoral T cell density on repeat biopsy; BBB permeability on perfusion MRI	Preclinical: patient-derived GBM organoid + TTFields + CAR-T co-culture; clinical: correlative substudy in ongoing CAR-T trials

**Abbreviations:** ADC, antibody-drug conjugate; AMPK, AMP-activated protein kinase; BBB, blood–brain barrier; BRCA1, breast cancer susceptibility gene 1; CAR-T, chimeric antigen receptor T cell; cGAS, cyclic GMP-AMP synthase; CRT, chemoradiotherapy; CSF, cerebrospinal fluid; DCE-MRI, dynamic contrast-enhanced MRI; GBM, glioblastoma; HR, homologous recombination; IFN, interferon; LC3-II, microtubule-associated protein 1A/1B-light chain 3-II; ndGBM, newly diagnosed glioblastoma; NK, natural killer; OS, overall survival; PARPi, PARP inhibitor; RT, radiotherapy; TAM, tumor-associated macrophage; TCR, T cell receptor; TME, tumor microenvironment; TMZ, temozolomide; TTFields, Tumor Treating Fields.

## Data Availability

No new data were created or analyzed in this study.
